# A Practical Application Combining Wireless Sensor Networks and Internet of Things: Safety Management System for Tower Crane Groups

**DOI:** 10.3390/s140813794

**Published:** 2014-07-30

**Authors:** Dexing Zhong, Hongqiang Lv, Jiuqiang Han, Quanrui Wei

**Affiliations:** School of Electronic and Information Engineering, Xi'an Jiaotong University, Xi'an 710049, China; E-Mails: lhqxinghun@live.cn (H.L.); jqhan@xjtu.edu.cn (J.H.); wquanrui@gmail.com (Q.W.)

**Keywords:** Internet of Things, wireless sensor network, safety management system, tower crane group, remote supervision

## Abstract

The so-called Internet of Things (IoT) has attracted increasing attention in the field of computer and information science. In this paper, a specific application of IoT, named Safety Management System for Tower Crane Groups (SMS-TC), is proposed for use in the construction industry field. The operating status of each tower crane was detected by a set of customized sensors, including horizontal and vertical position sensors for the trolley, angle sensors for the jib and load, tilt and wind speed sensors for the tower body. The sensor data is collected and processed by the Tower Crane Safety Terminal Equipment (TC-STE) installed in the driver's operating room. Wireless communication between each TC-STE and the Local Monitoring Terminal (LMT) at the ground worksite were fulfilled through a Zigbee wireless network. LMT can share the status information of the whole group with each TC-STE, while the LMT records the real-time data and reports it to the Remote Supervision Platform (RSP) through General Packet Radio Service (GPRS). Based on the global status data of the whole group, an anti-collision algorithm was executed in each TC-STE to ensure the safety of each tower crane during construction. Remote supervision can be fulfilled using our client software installed on a personal computer (PC) or smartphone. SMS-TC could be considered as a promising practical application that combines a Wireless Sensor Network with the Internet of Things.

## 1. Introduction

In recent decades, Wireless Sensor Network (WSN) technology has attracted wide attention from academia, industry and even governments. It quickly became one of the most promising core technologies in the military, health care [1], building structural monitoring [2], smart home systems [3,4], target tracking [5], space exploration [6], ocean monitoring [7], oil and gas exploration [8], underground mines [9], environmental monitoring [10] and other areas [11].

The concept of Internet of Things (IoT) can be traced back to 1999, and it was first proposed by the Auto-ID Center of the Massachusetts Institute of Technology (MIT) [12]. Utilizing information sensing equipment such as radio frequency identification (RFID), infrared sensors, global positioning devices and a variety of other sensors, according to the agreed protocol, any object can be connected to the internet for information exchange and communication. This network can fulfill a bunch of tasks, e.g., the intelligent identification of objects, positioning, tracking, monitoring and supervision. IoT has gained much attention from researchers and practitioners from around the world [13–17].

The concept of IoT has revolutionized traditional thoughts, which separate the physical infrastructure and IT infrastructure, whereas the IoT associates airports, buildings, roads, *etc.* with IT systems by cable, chip and broadband. IoT provides a “*data soul*” to the fundamental constructions of the so-called digitization of things. Within IoT networks, economic management, social management, production and even personal daily life will be more convenient and effective.

Herein we introduce the concept of IoT in the construction industry field, and develop a practical application that combines WSN and IoT. In the practical construction worksite, the safety issues of tower crane groups within overlapping working areas have attracted considerable attention in the field of academia and industry [18–21]. After the optimal layout [22,23] of a tower crane group in the initial phase, a dynamic safety management system has become a very urgent need to ensure safety during the construction, especially in the large-scale overlapping working areas. Different from single crane safety issue [24,25], the high-speed stable communication, accurate real-time signal processing and effective anti-collision algorithms are core problems dealing with the safety and collaboration issues of tower crane groups. Furthermore, for all tower crane groups in a certain city or province, conventional regulatory approaches for authorities are inspections and spot checks, which cannot obtain the real-time status of each group and maintain long-term monitoring throughout the duration of operation.

The emerging Internet of Things (IoT) [13,16,26] provides a feasible and effective solution for the abovementioned safety issues and regulatory challenges. Our proposed Safety Management System for Tower Crane Groups (SMS-TC) can be considered as a specific application of IoT [12] in the construction industry field, which belongs to both the industrial IoT and local IoT.

According to the typical architecture of IoT [27,28], a simple and intuitive classification of IoT is divided into three layers:

- Perception Layer [29,30], the skin and facial features of IoT for object recognition and information collection;
- Network Layer [31,32], the nerve centre and brain of IoT for information transmission and processing;
- Application Layer [33,34], the so-called social division of labor (SDL) of IoT for specific needs of different industries to achieve a wide range of intelligent world or smart world.

**Figure 1.**
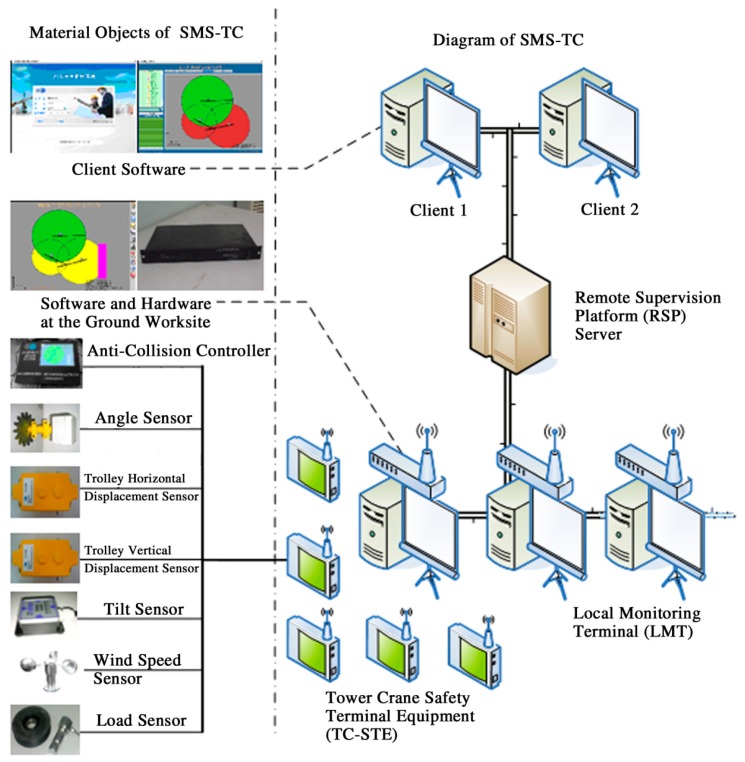
The system architecture diagram of safety management system for tower crane groups (SMS-TC).

Analogous to the above three-layer architecture, the three main components of SMS-TC are deemed to belong to each layer respectively. The system architecture diagram of SMS-TC is presented in Figure 1.

For individual tower cranes, the status information of trolley, jib and tower can be detected by a set of customized sensors. To measure the position of the trolley, two displacement transducers [35] are used for the horizontal and vertical directions. To measure the position of the jib, an angular displacement transducer is used. To detect the status of tower body, load, tilt [36] and wind speed suitable sensors are applied for the integrated information. All the results from sensors are transmitted through signal lines to a central control node, which is installed in the driver's operating room. Since all sensors are installed on the tower within 10 m distance from the central node, they can be connected via the wired way.

For tower crane groups within an overlapping working area, there could be more than three cranes in the work area, making it very difficult for the driver to pay attention to all the other adjacent cranes. Therefore, the status of the whole group needs to be transmitted to each tower crane for the anti-collision algorithm. Subject to the complex environmental factors of worksites [37,38] and the passage of construction vehicles and workers, it would be inconvenient and expensive to transmit information among a tower crane group in a wired way. Therefore, wireless communication [2,39–41] is a necessary alternative in this particular situation, which can avoid interruptions during the construction process and realize relatively stable communication between the tower crane group.

Through the signal lines, the Tower Crane Safety Terminal Equipment (TC-STE) acquires data from all sensors installed at the key parts of tower cranes. Meanwhile, TC-STE reports the real-time status of each tower crane to a Local Monitoring Terminal (LMT) through a wireless network. TC-STE belongs to Perception Layer and Part 1 of the Network Layer.

The main functions of LMT are to act as a digital repeater for each TC-STE, as data flow controller, and as implementer of the wireless network protocol. Meanwhile, LMT is used to communicate with the Remote Supervision Platform (RSP) server. LMT belongs to Part 2 of the Network Layer, which is the core device of the Network Layer.

RSP acquires data from LMT in each worksite through the General Packet Radio Service (GPRS) [42,43] or the third-generation mobile (3G) Internet [44]. Supported by a dedicated server, RSP can be easily connected via the Internet using our client software. Supervisors can register a tower crane and determine its real-time status as well as historical data of all the tower cranes in a certain area. RSP belongs to Application Layer and Part 3 of the Network Layer.

Additionally, different from the typical three-layer architecture of IoT, SMS-TC has a fourth layer: the Supporting Layer, which can fulfill the thinking, identifying and decision-making tasks as a functional brain of the IoT. The core component of the Supporting Layer is the Anti-Collision Controller (ACC) for the individual tower cranes, which is the original purpose of this research.

As a processing device, ACC is integrated in TC-STE that is directly connected to various sensors via wired links. Therefore, stable and high-speed transmission can be fulfilled. Raw data are transmitted from sensors to TC-STE by the short wired network; processed raw data are transmitted from the TC-STE to the LMT by a wireless network; processed data are transmitted from the LMT to the RSP by GPRS or 3G Internet. As a result, the total amount of data transmission of the SMS-TC is reduced significantly in this wired plus wireless transmission method. Furthermore, the energy consumption for the transmission is reduced and the energy conservation of the system is improved, especially for the wireless equipment.

One of our innovations is the functional sensor network consisting of the short wired signal line, short wireless Zigbee network, long wireless GPRS and Internet. The other is the way of data transmission, *i.e.*, each TC-STE has the capability of processing raw data, which can significantly reduce the total amount of data.

The rest of the paper is organized as follows: the sensor network and anti-collision algorithm for tower crane groups are given in Section 2. The proposed dynamic Safety Management System for Tower Crane Groups (SMS-TC) is described in Section 3. The application of SMS-TC is presented in Section 4. Finally, Section 5 provides our conclusions.

## 2. Sensor Network and Anti-Collision Algorithm for Tower Crane Group

### 2.1. Sensor Network

**Figure 2.**
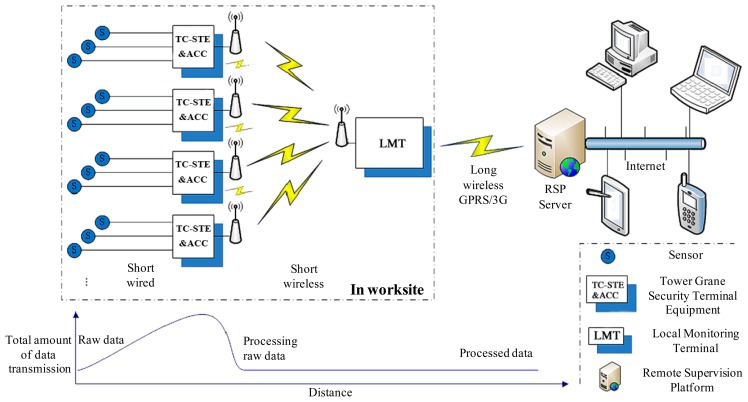
Sensor network and data transmission of safety management system for tower crane groups (SMS-TC). LMT, Local Monitoring Terminal; TC-STE, Tower Crane Safety Terminal Equipment; ACC, Anti-Collision Controller; RSP, Remote Supervision Platform.

As a typical practical application of IoT in the field of construction industry, SMS-TC has a typical comprehensive sensor network, which consists of a short wired signal line, short wireless Zigbee [45] network, long wireless GPRS/3G communication and Internet, as shown in Figure 2.

In order to detect the operating status of each tower crane and its trolley, six sensors were employed and directly connected to TC-STE via a short wired signal line. They are the horizontal and vertical displacement sensors for the trolley, the angle sensor for the jib, and the load, tilt and wind speed sensors for the tower, respectively. The wired signal lines can guarantee the stability of high-speed transmission for the raw data obtained from these sensors. ACC was integrated in TC-STE to process the raw sensor data. Only processed raw data were transmitted to the LMT via the short wireless Zigbee network.

A star topology [45] was applied in this Zigbee network with LMT as the coordinator. Each TC-STE can receive the operating status of other tower cranes from the LMT. Furthermore, each TC-STE is a node with routing function. Due to the presence of obstacles or the poor propagation conditions, some nodes may not be able to maintain proper communication with the LMT; these routing nodes play a key role in maintaining the functional network. Usually, we place LMT in a temporary shed at the worksite. The LMT antenna is installed on the shed roof. The TC-STE antenna is installed outside of driver's operating room that is on the top part of the tower. As a result, the wireless communication among each TC-STE is good and without obstacles. In addition, it ensures that more than one TC-STE can be in the line of sight (LOS) with the LMT, which can be satisfied in almost all construction sites. With functional routing nodes, the wireless network with star topology is very robust and it can meet the requirements of practical applications.

To avoid the interferences due to multiple simultaneous communications, our solution is to apply a broadcast and response mechanism. LMT cyclically broadcasts the data acquisition requests for each TC-STE node, and the transmission interval is adjustable based on the number of TC-STEs in a certain WSN. As a result, at each moment only one TC-STE sends its operating status information to the LMT, which has a full duplex communication function. Thus, ACC can implement the anti-collision algorithm according to the real-time status of the whole group. Moreover, the amount of raw data processing can be significantly reduced for the subsequent wireless communications. As a result, the total amount of data transmission is substantially reduced as well.

In each worksite, the LMT can gather all of the measured data values of a tower crane group and alarm information, and then the information is transmitted to the RSP server at the monitoring control center through GPRS or 3G Internet. As a typical representative of the “2.5G” mobile technologies, GPRS is a developed bearer service for the global system for mobile-communications (GSM) that greatly improves and simplifies wireless access to packet data networks [46]. The advantages of GPRS can be summarized as the improvement of the radio resource utilization, the volume-based billing instead of the “online” time, higher transfer rates, shorter access times, and the simplified access to packet data networks, e.g., to the Internet. The latter, 3G, refers to the collection of third-generation mobile technologies, which would be the first choice for upgrading the SMS-TC.

For supervisors and clients of SMS-TC, the RSP server can be easily connected via the Internet using our client software. The historical and real-time data of each tower crane at each worksite can be obtained from the RSP server, as long as the user has sufficient authority and Internet access. Regardless of time and space, administrators can check any desired information in this IoT system using PCs, notebooks, tablets, or mobile phones.

The short wireless Zigbee network is not based on Internet Protocol (IP); instead, it is based on the IEEE 802.15.4 protocol. The long wireless GPRS and Internet are based on Transmission Control Protocol/Internet Protocol (TCP/IP). A Subscriber Identity Module (SIM) card is installed in the LMT to ensure GPRS communication with the RSP. Particular IP addresses are assigned to each LMT and RSP, their communication port is 5000. The PC and smartphone with our client software are linked to the RSP server through port 2500.

As shown in Figure 2, the networks of SMS-TC consist of the short wired signal line, short wireless Zigbee network, long wireless GPRS and Internet. A set of customized sensors are utilized to obtain the real-time status of each tower crane. WSN is applied to fulfill wireless communication between tower crane group and LMT at the ground worksite. To facilitate remote supervision, LMT records the data and reports them to RSP through GPRS. Remote supervision can be implemented using our client software installed on PC or smartphone. RSP is the key component to make SMS-TC a meaningful practical application of IoT. In this case, we think that SMS-TC is beyond the concept of Machine-To-Machine or Wireless Sensor Network (M2M/WSN). Therefore, we could consider SMS-TC as a practical application that demonstrates the promising prospects of WSN and IoT in the construction industry.

### 2.2. The Customized Network Protocol of Wireless Sensor Network (WSN)

At the local worksite, a customized WSN protocol is developed based on the IEEE 802.15.4 protocol. The data transmitted within the WSN can be divided into two categories: the static data and the dynamic data. The former includes the static information of the worksite (site ID, site name, network configuration, safety responsibility, *etc.*), the static information of the equipment (model of tower cranes, number of tower cranes, relative coordinates, lengths of front and back jibs, heights of tower and jib, *etc.*), and the configuration information of tower cranes (optional functions, display brightness, audio volume, sensor calibration, forbidden zones, working area, *etc.*). The latter includes the calling of LMT and the response of each tower crane. Generally, the static data are used to exchange information in the initial installation and equipment maintenance phases, while the dynamic data are usually used in the anti-collision operational phase, wherein, the static data have a higher priority than the dynamic data.

The static data are transmitted by the client software installed in the LMT. To ensure that the critical information is sent correctly, a retransmission mechanism is adopted when the feedback is missing or an error ocurrs. If the LMT does not receive an appropriate feedback from a specific TC-STE within 10 s, the client will automatically prompt a manual retransmission of the data by the personnel.

For the dynamic data, there is no need to have a retransmission mechanism in our practical application. By using the broadcast and response mechanism, the LMT cyclically broadcasts the data acquisition request of each TC-STE. After receiving the request, each TC-STE will compare the target information of the request with itself. Only the requested TC-STE needs to broadcast its real-time status information into the network. If LMT does not receive the corresponding response before sending the next request for another TC-STE, there is no need to wait; instead, the LMT will send the next request directly. Such a mechanism is conducive to ensuring the implementation of our anti-collision algorithm for the running TC-STEs, the hidden terminal (the shutdown TC-STE) does not affect the remaining running TC-STEs. According to the descriptions of star topology, routing nodes and specific setting of antennas in the above subsection, the wireless network is considerably robust. As a result, this mechanism is suitable for the complex conditions of a construction site.

**Figure 3.**
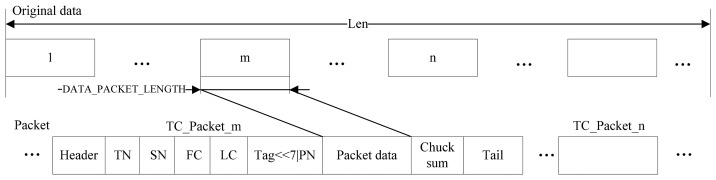
General frame structure of the data transmitted within WSN. Len presents the length of data; the sender takes use of “DATA_PACKET_LENGTH” to split the data into ┌ Len/DATA_PACKET_LENGTH┐ TC_Packets. For each TC_Packet, Header and Tail are the head and tail information, respectively. TN represents the netID of source node; SN represents the netID of target node; FC represents the data type; LC represents the directive No.; Tag is the last TC_Packet; PN represents the serial number of multiple TC_Packets; Chuck sum is check information. The receiver restructures the packets according to PN, and it can recover the original data.

How to choose the frequency of broadcast cycle of the LMT is a critical issue in the wireless network, since all the data communication within the network is coordinated by the LMT. The transmission interval is adjustable based on three main aspects as follows: (1) the number of TC-STEs in a specific wireless network; (2) the real-time requirements of the anti-collision algorithm; (3) the loss of wireless modules in a practical case. Concretely, the transmission interval varies between 30 and 150 ms, which can be divided into 10 levels. The frequency of broadcast cycle is empirically chosen according to working conditions and the instruction guide. The general frame structure of the data is depicted in Figure 3.

The length of static data, which varies between 12 B to 3 KB, is related to the data type and the number of tower cranes in the network. The length of dynamic data is fixed as 16 B. Both static and dynamic data are transmitted using the general packet with 25 B, as shown in Figure 3. The transmission rate of Zigbee is about 20–30 Kps; without considering the reaction time of threads and the routing time, the sending time for static data is less than 1.2 s, and the one for dynamic data is almost negligible.

### 2.3. Anti-Collision Algorithm

According to a comprehensive analysis of the data collected by a variety of sensors, TC-STE can implement the appropriate alarms and preventive functions, including collision avoidance of tower cranes against other adjacent cranes and buildings, protection of forbidden regions, reminding of lifting area, protection of tilt overrun, wind speed overrun and moment overload.

**Figure 4.**
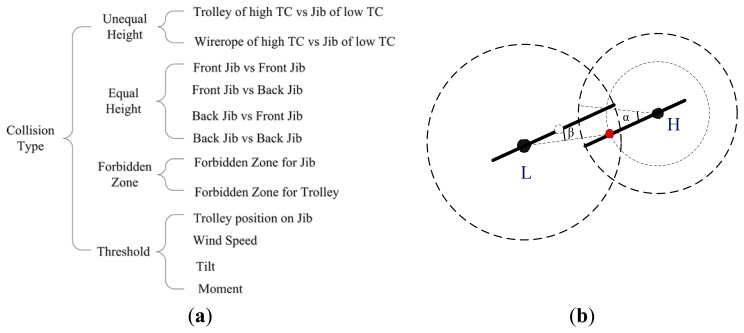
(**a**) The classification of collision types; and (**b**) A typical collision case: schematic plan of collision between trolley of high tower crane and jib of low tower crane. L and H represent the centers of low-jib tower and high-jib tower; α and β represent the angles of rotation; the white and red circles represent the trolleys of low-jib tower and high-jib tower, respectively.

One of the core functions of the TC-STE is the avoidance of collisions against other adjacent tower cranes, which is performed by the integrated Anti-Collision Controller (ACC) according to the status of the whole group. The core of our proposed anti-collision algorithm is to find all the collision conditions that need to be avoided during operation. The anti-collision algorithm takes a variety of complex situations into consideration, including each tower crane, the buildings under construction, forbidden zones and weather conditions. The real-time status of each tower crane is obtained by a set of customized sensors located in different parts of the tower cranes. The classification of all the collision types is provided in Figure 4a. Moreover, we take a typical collision, shown in Figure 4b, as an example to demonstrate our proposed anti-collision algorithm.

As shown in Figure 4b, two tower cranes have an overlapping working area. Generally, in order to increase the operating range of tower cranes within overlapping working area, the heights of their jibs are set to be different. *H* represents the tower crane with the higher jib, and *L* refers to the one with a lower jib. The coordinates of tower *H*, the height and length of its jib are given as (*x_H_*, *y_H_*), *h_H_*, and *r_H_*, while the coordinates of tower *L*, the height and length of its jib are given as (*x_L_*, *y_L_*), *h_L_*, and *r_L_*. The coordinates of tower crane and the length of its jib have been identified when it was installed. The height of the jibs increases gradually during the building process.

In this situation, there is a risk of collision between the trolley of the high tower crane and the jib of the low one. Based on the three sensors, *i.e.*, the horizontal and vertical position sensors for trolley as well as the angle sensor for jib, three real-time parameters of the tower cranes can be obtained. For tower crane *H*, the angle of jib α*_H_*, the location of trolley *L_H_* and the height of trolley *T_H_* can be obtained.

If *T_H_* > *h_L_*, there is no chance for the collision of two tower cranes.

Otherwise, if *T_H_* ≤ *h_L_*, there is a risk of collision. An early warning function can be generated by calculating the distance from the trolley of *H* to the jib of *L*, *i.e.*, distance from a point to a line. The trolley coordinates of *H* can be expressed as (*x_H_*+*L_H_* cos(α*_H_*), *y_H_*+*L_H_* sin(α*_H_*)), the jib equation of *L* as follows:

(1) $$y-y_{L}=\left(x-x_{L}\right)\tan\left(\alpha_{L}\right)$$

where α*_L_* is the jib angle of *L*. Then the distance from the trolley of *H* to the jib of *L* can be calculated as follows:

(2) $$d_{1}=\frac{\left|\left(x_{H}+L_{H}\cos(\alpha_{H})-x_{L}\right)\tan(\alpha_{L})-\left(y_{H}+L_{H}\sin(\alpha_{H})-y_{L}\right)\right|}{\sqrt{\tan^{2}(\alpha_{L})+1}}$$

The distance from the trolley of *H* to the coordinates of tower *L* can be calculated as follows:

(3) $$d_{2}=\sqrt{{\left(x_{H}+L_{H}\cos(\alpha_{H})-x_{L}\right)}^{2}+{\left(y_{H}+L_{H}\sin(\alpha_{H})-y_{L}\right)}^{2}}$$

We set a safe distance Δ, an if *d*_2_ ≤ *r_L_* and *d*_1_ ≤ Δ, a warning message is issued. This is one of the typical collision conditions. The core of our anti-collision algorithm is to find all potential collision risks, as shown in Figure 4a, and then it alarm in advance correspondingly, even taking necessary protective measures, such as power-off protection in a specific direction.

Using the algorithm described above, the anti-collision algorithm of the whole tower crane group can be executed according to the coordinates of each tower in the group, the height and length of its jib, and the location of its trolley. All this information is shared by the LMT via Zigbee wireless networks.

As presented in Figure 4a, we have classified all the types of potential collisions. The collision between the trolley or wirerope of a high tower crane and the jib of a low tower crane is the most typical and representative example, other collisions listed in Figure 4a can be solved by similar approaches. The most important tasks are to find the conditions of potential collisions and the core of our anti-collision algorithm is to avoid all these conditions.

Another risk of tower crane is moment overload. If the moment exceeds the rated value, the crane will be imbalanced, which can cause serious accidents. The unit of moment for a tower crane is *Ton* × *Meter*. Given the location of trolley *L* and the weight of the load *M*, the moment can be written as:

(4) F=L×M

We compare *F* with the rated value, and if it exceeds 90% of the rated value, an alarm will be issued. In this case, the trolley cannot move forward and the braking controller will be activated. The other two preventive functions are designed for tilt and wind speed overruns.

To summarize, the horizontal and vertical displacement sensors for the trolley as well as the angle sensor for the jib can provide the real-time pose of individual tower cranes. Each TC-STE reports relevant information to the LMT that is the coordinator of the wireless network, so that the status information of the whole tower crane group can be shared to facilitate implementation of the anti-collision algorithm. The load sensor and horizontal displacement sensor of the trolley can be used to calculate the moment, thus preventing the jib imbalance. Tilt and wind speed sensors are used for other alarms, respectively.

Compared with the computation times and subsequent transmission times of data within the WSN, the movements of trolley and jib are relatively slow. The total delay of alarm is less than 0.4 s, and the power protection delay is less than 0.5 s. There are three steps to avoid potential collision risks: first, when the jib moves into the area where a collision may occur, the relevant area will turn yellow in the screen. Second, when the jib or trolley is 8 m away from potential collision, the TC-STE will alert and the relevant area will turn red in the screen. Third, if still without proper operation, when they are only 4 m away from collision, operation in this direction will be put under power-off protection, while the other five directions are unaffected. This condition has taken into account the inertia factor.

## 3. Safety Management System for Tower Crane (SMS-TC)

The building construction is implemented by the cooperation among various building machinery and workers, in which the tower crane group is one of the most important modern machinery for industrial and civil construction. Therefore, the safety management of the tower crane group is an essential issue for building projects. Our proposed SMS-TC can be considered as a targeted application of IoT in the building industry.

### 3.1. Core Components of SMS-TC

The material objects of SMS-TC can be found in Figure 1.

**Figure 5.**
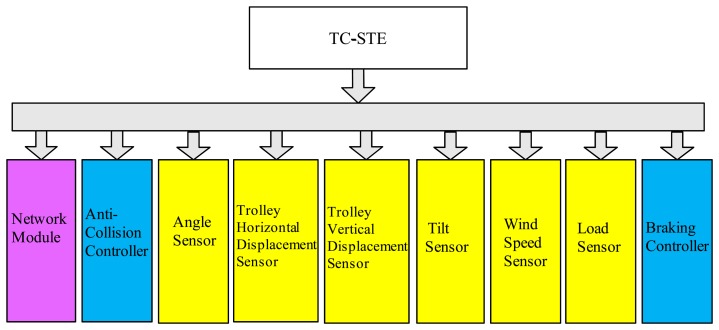
Main components of safety management system for tower crane (TC-STE). Purple box represents the network module; blue boxes represent controllers; yellow boxes represent sensors.

**Figure 6.**
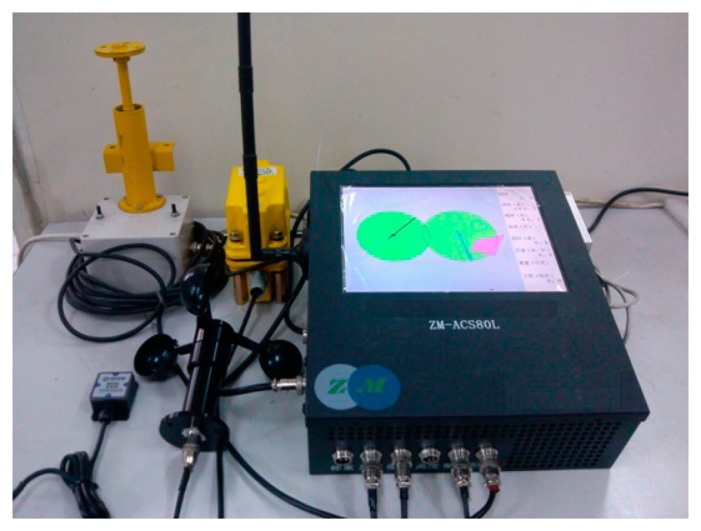
Tower crane safety terminal equipment (TC-STE) and the set of customized sensors.

**Figure 7.**
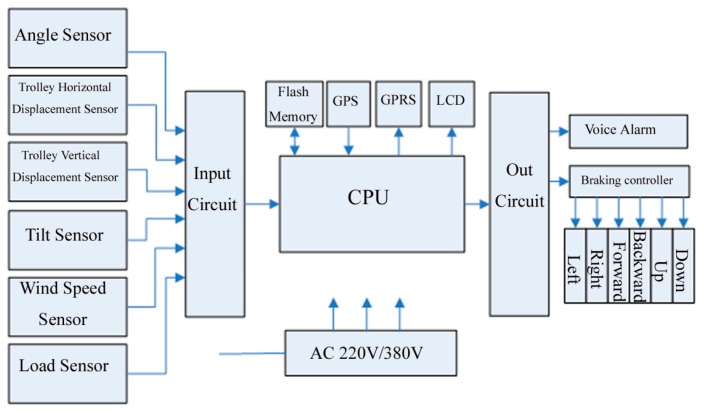
Functional block diagram of safety management system for tower crane (TC-STE).

- Tower crane safety terminal equipment
  TC-STE belongs to Perception Layer and Network Layer Part 1 of IoT. The main components of TC-STE are presented in Figure 5.
  The network module's function is bidirectional communication with the LMT. It reports the real-time status of individual tower cranes to the LMT, which includes the position of the trolley, the angle of the jib, and the load, tilt and wind speed sensor information. Meanwhile, it receives the real-time status of other adjacent tower cranes from the LMT. The anti-collision controller processes the raw data of the six special sensors and runs the anti-collision algorithm. The anti-collision controller (ACC) is the core component of the fourth layer of IoT, *i.e.*, the Supporting Layer, which can fulfill the thinking, identifying and decision-making tasks as the functional brain of the IoT system In addition, it can substantially reduce the total amount of data transmission in the whole system.
  TC-STE acquires data from sensors at the key parts of tower crane, and then it can save and analyze these data, while it communicates through a wireless network to report the real-time status. According to the shared information of the LMT, the TC-STE can implement the alarming and braking tasks. For more details of the design and implementation of TC-STE, the physical picture and corresponding functional block diagram of the TC-STE are provided in Figures 6 and 7, respectively.
- Local monitoring terminal
  LMT belongs to Network Layer Part 2 of the IoT. The main purposes are to act as the digital repeater for each TC-STE, the data flow controller, the implementer of the wireless network protocol. Meanwhile, LMT is used to communicate with the Internet for data interaction, which is the core device of the Network Layer of IoT.
- Remote supervision platform
  The RSP belongs to the Application Layer of the IoT and Part 3 of the Network Layer. RSP is an essential component of our proposed SMS-TC. In addition, RSP is the key component to make SMS-TC a meaningful practical IoT application. Technically supported by a dedicated data server, the official supervisors can easily log in to the RSP via the Internet to approve and register a certain tower crane before construction. During construction, they can gain the real-time and historical status information of every individual tower crane in their area of jurisdiction. After construction, they can obtain a comprehensive statistical data report of the entire process.

### 3.2. SMS-TC Softwares

The entire software platform consists of three parts: the local client side (LCS), the intermediate server side (ISS), and the remote client side (RCS).

**Figure 8.**
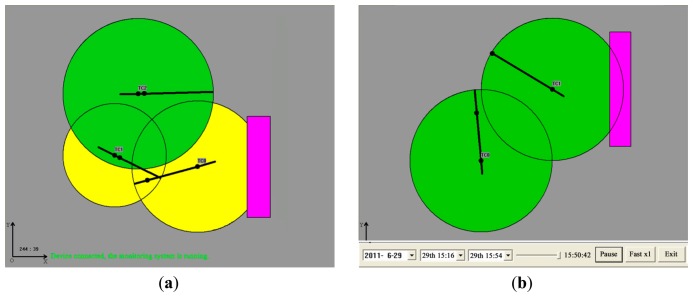
(**a**) Real-time monitoring of local client side (LCS); and (**b**) Historical data playback.

**Figure 9.**
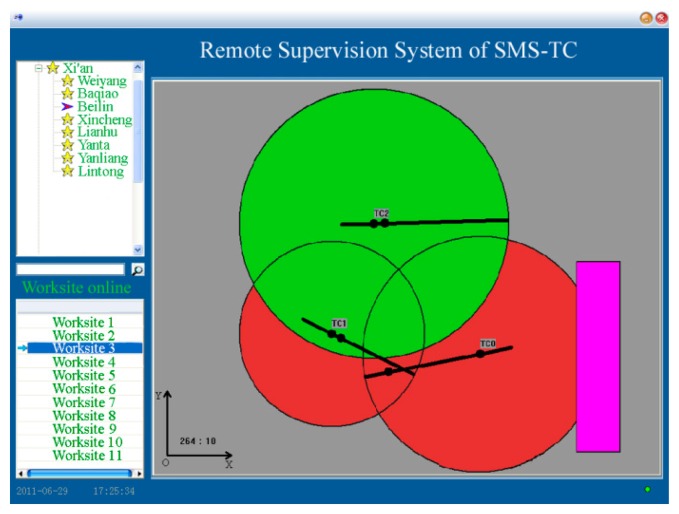
Real-time monitoring interface of remote client side (RCS).

- Local client side
  LCS is adopted to acquire the status of each tower crane through a wireless network and display the running graph of the whole tower crane group. Moreover, LCS makes the timely decisions to alarm or even brake before any potential collision. Figure 8 presents the displays of LCS. Real-time and historical data can be displayed by the LCS.
- Intermediate server side
  The main purpose of the ISS is to manage the massive amounts of data collected from each individual building project site. The dedicated server stores the whole database. ISS is the data-managing intermediary between the LCS and RCS.
- Remote client side
  The users are divided into different levels with different permission to access the database in ISS. The supervisor has the highest authority. Figure 9 presents the RCS displays.

After login, the user can select a city and a specific building site via a menu. All the registered tower cranes can be found with the project information, tower crane configuration, real-time data, real-time video signals as well as the historical data, alarm times and so on. The statistical information can be generated to facilitate supervision.

## 4. Application of Safety Management System for Tower Crane (SMS-TC)

### 4.1. Target Tower Crane Group

**Figure 10.**
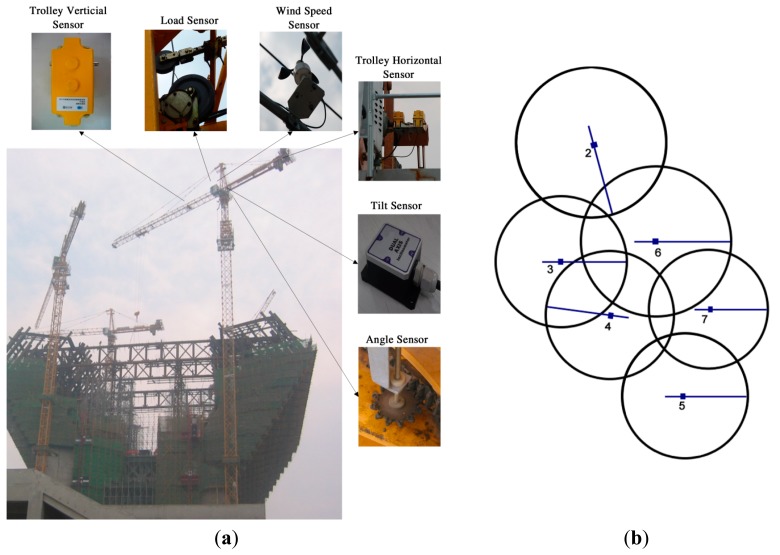
(**a**) The tower crane group in the worksite of Famen Temple Dagoba (FTD) and the actual assembled positions for six types of sensors; and (**b**) Schematic plan of a tower crane group. Number 2-7 represent six tower cranes with their netIDs.

A real-time safety management system for tower crane groups was applied to a large-scale complex building worksite (Famen Temple Dagoba (FTD) in Baoji, Shaanxi Province, China) during its construction. FTD is like two hands clasped together with the height of 148 m, which is equivalent to a fifty-storey building. Figure 10 illustrates the practical construction process of FTD. There are six tower cranes as a group within an overlapping working area.

Also shown in Figure 10a, six types of sensor were applied for measuring the real-time status of tower, jib and trolley. Taking tower crane No. 4 as an example, the actual assembled positions of sensors are pointed out as well. Two displacement sensors are used to measure the horizontal and vertical position of the trolley. An angle sensor is used to measure the angle of the jib. The load sensor is used to measure the loading weight. The wind speed and tilt sensors are involved as well. In addition, the schematic plan of the tower crane group is shown in Figure 10b.

According to the pre-scheduled commands of the tower crane safety terminal equipment (TC-STE), the sensors automatically and continuously measure the actual data of each individual tower crane. All the data are processed by the TC-STE and transmitted to the Local Monitoring Terminal (LMT) on the ground through a wireless network. Table 1 provides the specifications for the six types of sensors.

The communication network architecture of SMS-TC at FTD is designed exactly as the sensor network shown in Figure 2. LMT is a coordinator of the sensor wireless network as well as a communicator with the remote supervision platform (RSP) through GPRS.

### 4.2. Results of SMS-TC

**Figure 11.**
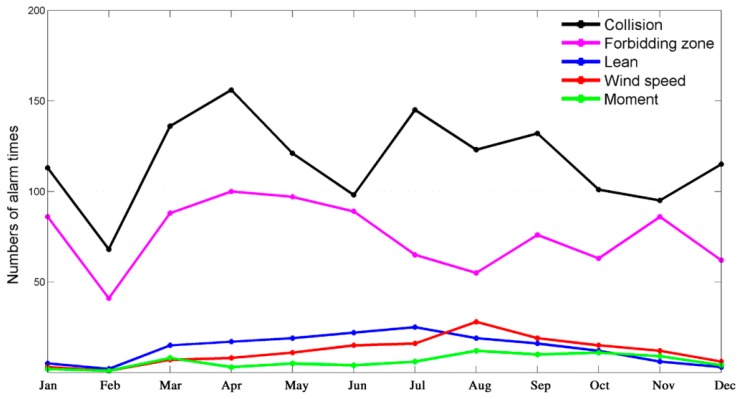
The number of each alarm times during the year 2013.

We record five types of alarm during the construction process, which include collision alarms, forbidden zone alarms, tilt alarms (or lean alarms), wind speed alarms and moment overload alarms. Figure 11 illustrates the number of alarms in each month during a year. Collision and forbidden zone alarms are the two most frequent types of alarms during the construction process, while the other three alarms are considerably less frequent.

Collision is the main risk in a tower crane group within overlapping working areas, therefore, the collision alarm is the fundamental function and service of our proposed SMS-TC to remind the drivers. Forbidden zone alarms arethe second most important function of SMS-TC to ensure that the jib and trolley of a tower crane cannot enter all the forbidden zones, such as buildings, roads, and so on. Besides the two abovementioned main functional alarms, SMS-TC can also provide other three alarms, including tilt, wind speed and moment alarms, respectively. Since the rated value of the moment of a tower crane is relatively large enough, carrying materials and components seldom causes alarms.

As shown in Figure 11, these three alarms are relatively few compared with collision and forbidden zone alarms. There are significant declines of each alarm frequency in February since the spring festival holidays are celebrated in this month in China.

Directly obtained from RSP, the statistical results of the six tower cranes are presented in Table 2. Alarms by each TC-STE are recorded and divided into the following categories: alarm against building under construction (BUC), alarm against given threshold (GT), alarm against collision with each other, alarm against forbidden zone (FZ), and the number of power protections (PP).

**Figure 12.**
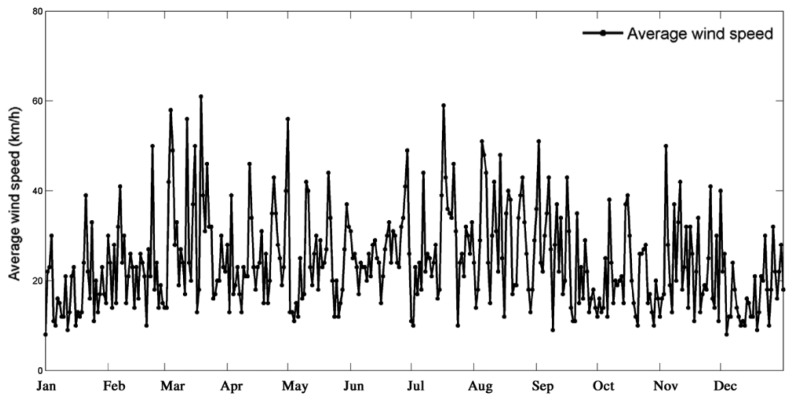
The average wind speed during the year 2013.

In addition, utilizing the wind speed sensor, the SMS-TC can measure the wind speed in real-time and provide the average one during a day. Figure 12 depicts the average wind speed during the construction process. In the future, according to the data collected by load sensors, we can calculate the total weight of each tower crane in each day, so that the efficiencies of all tower cranes can be achieved to evaluate the work while it can also be utilized to improve the optimal layout of tower crane groups as an important reference.

## 5. Conclusions

In this paper, we have proposed a practical application combining a Wireless Sensor Network (WSN) and the nternet of Things (IoT) in the construction industry field, which was developed and named as Safety Management System for Tower Crane Groups (SMS-TC). The core components and softwares of SMS-TC were presented, respectively. The three main components could be roughly classified in the three layers of the IoT: Perception Layer, Network Layer and Application Layer. Additionally, as a supplement of the typical three-layer architecture of IoT, the SMS-TC has a fourth layer: Supporting Layer, which can fulfill the thinking, identifying and decision-making tasks as a functional brain of the IoT. The SMS-TC sensor networks consist of a short-distance wired signal line, short-distance wireless Zigbee network, long-distance wireless GPRS and Internet. The advantages of this comprehensive network structure include that the total amounts of data transmission are significantly reduced in the wireless transmission phase, the energy consumption for the transmission is reduced and the energy conservation of the system is improved as well. A set of customized sensors are utilized to obtain the complex real-time status of each tower crane. The WSN is used to fulfill wireless communication between each tower crane and the Local Monitoring Terminal (LMT) at the ground worksite, which can avoid interruptions during the construction process and realize high-speed stable communication among the tower crane group. Based on the global status data of the whole group, an anti-collision algorithm was executed to ensure the safety of each tower crane during construction. In order to facilitate remote supervision, LMT records the real-time data and reports them to the Remote Supervision Platform (RSP) through GPRS. Remote supervision can be fulfilled using our client software installed on a PC or smartphone. As a system with mechanics, electrics, algorithms and network communications all in one, SMS-TC could be considered as a practical application that demonstrates the promising prospects of WSN and IoT in China's building industry and engineering in the near future.

## Figures and Tables

**Table 1.** Sensor specifications.

Parameter	Sensor				

	Trolley Sensor	Angle Sensor	Tilt Sensor	Load Sensor	Wind Speed Sensor
Measurement Range	0–100 m	0°–360°	0°–15°	2–50 t	0–40 m/s
Degree of Precision	0.1 m	0.18°	0.05°	0.05%	0.1 m/s
Temperature Range	−20°–60°	−20°–60°	−40°–85°	−20°–60°	−20°–60°

**Table 2.** Statistical results of the six tower cranes.

No. of TC	Alarm (BUC)		Alarm (GT)			Alarm Against Collision with Each Other						Alarm (FZ)				No. of PP
			
	Margin	Height	Tilt	Wind	Moment	Left	Right	Forward	Backward	Up	Down	Left	Right	Forward	Backward	
2	110	0	1	12	0	841	394	3	0	0	0	20	7	27	0	742
3	1771	0	1	7	0	359	327	2	0	0	0	2133	362	2494	1	4020
4	21	209	6	6	0	199	68	266	0	0	0	22	206	228	0	73
5	20	13	179	70	0	660	531	1185	3	0	0	55	34	89	0	474
6	7	186	2	667	77	462	541	925	78	0	1003	163	31	194	0	608
7	255	145	0	368	842	623	336	0	0	0	0	478	125	603	0	1888

Notes: TC = Tower Crane, BUC = Building Under Construction, GT = Given Threshold, FZ = Forbidden Zone, PP = Power Protections.
